# A novel de novo *AP2M1* variant in a patient with attention‐deficit/hyperactivity disorder, oppositional defiant disorder, and unexpected hemiplegic migraine

**DOI:** 10.1111/head.15017

**Published:** 2025-07-28

**Authors:** Giuseppe D. Mangano, Vincenzo Antona, Giuseppe Santangelo, Gabriele Di Pasquale, Jacopo Colella, Vincenzo Salpietro, Giuseppa R. Mangano, Vincenzo Raieli

**Affiliations:** ^1^ Department of Medicine and Surgery University of Enna Kore Enna Italy; ^2^ Department of Health Promotion, Mother and Child Care, Internal Medicine, and Medical Specialties “G. D'Alessandro” University of Palermo Palermo Italy; ^3^ Neurology and Psychiatry Division, Pediatric Hospital “Giovanni Di Cristina” Azienda di Rilievo Nazionale ad Alta Specializzazione Civico Di Cristina Benfratelli Palermo Italy; ^4^ Pediatrics Unit, Department of Biotechnological and Applied Clinical Sciences University of L'Aquila L'Aquila Italy; ^5^ Department of Neuromuscular Disorders University College of London Institute of Neurology London UK; ^6^ Department of Psychology, Educational Sciences, and Human Movement University of Palermo Palermo Italy

**Keywords:** adaptor‐related protein complex 2 subunit mu 1, attention‐deficit/hyperactivity disorder, hemiplegic migraine, migraine genetics, neurodevelopmental disorder, oppositional defiant disorder

**Abbreviations**
ADHD: attention‐deficit/hyperactivity disorder
CME: clathrin‐mediated endocytosis
EEG: electroencephalography
GDD: global developmental delay
HM: hemiplegic migraine

The adaptor‐related protein AP2 is a heterotetrameric complex consisting of α, β, μ, and σ subunits, which plays a crucial role in clathrin‐mediated endocytosis (CME) and especially in the retrieval of synaptic vesicles at nerve terminals.^1^ Interestingly, several studies have elucidated that CME of plasma membrane proteins controls the neuronal surface expression of γ‐aminobutyric acid and glutamate receptors involved in long‐term plastic changes in neurotransmission and excitatory/inhibitory balance.^1^ However, only five individuals with *AP2M1* mutations associated with neurodevelopmental disorders have been reported in the literature so far,^1^, ^2^ and the few clinical data available suggest that *AP2M1* mutations cause a variable phenotype ranging from severe intellectual disability, autism spectrum disorder, and epilepsy to isolated global developmental delay (GDD). We report an individual carrying a novel de novo *AP2M1* variant with attention‐deficit/hyperactivity disorder (ADHD), oppositional defiant disorder, and unexpected hemiplegic migraine (HM).

This study was approved by the ethics committee Palermo 1 of “Paolo Giaccone” University Hospital of Palermo, Italy, and written informed consent for publication was obtained from the patient's parents. The proband, a 10‐year‐old male, is the only offspring born to healthy unrelated parents with an unremarkable family history. The proband's personal prenatal and perinatal history was uneventful, and his developmental milestones were regular. From an early age, he was very active and unable to regulate his behavior, resulting in difficulties in integrating and in his school performance.

On admission at the Child Neuropsychiatric Department, the patient, aged 7 years 8 months, showed triangular facies and protruding ears; his head circumference, height, and weight values were at the 50th percentile. The neurological examination showed mild joint hyperextensibility, clumsiness, and normal deep tendon reflexes and cranial nerves.

The cognitive and behavioral assessment revealed fluent and structured speech but with difficulty in narrative, severe deficits in behavioral regulation, including restlessness and poor ability to wait and follow rules, and poor emotional control with aggressive behavior.

He is currently in the fifth grade, requiring individualized teaching.

At 7 years of age, the intellectual assessment with the Wechsler Preschool and Primary Scale of Intelligence, 3rd edition documented a nonhomogeneous cognitive profile (Total Intelligence Quotient [IQT] = 78), with performance skills (Performance Intelligence Quotient [IQP] = 93) as strengths, whereas verbal skills (Verbal Intelligence Quotient [IQV] = 74) and the processing speed index (Processing Speed Index [PSI] = 74) were weaknesses.

The Conners' Teacher Rating Scale, used to measure behavioral problems associated with ADHD, revealed high scores in all areas explored: oppositional scale T‐score = 72, cognitive problems/inattention T‐score = 83, hyperactivity T‐score = 79, “ADHD Index” score T‐score = 80.

At the age of 9 years 8 months, he had a HM following a fall with mild occipital head trauma. The HM was characterized by headache lasting several hours, associated with short‐lasting right body weakness (arm strength value of 3/5 on the Medical Research Council scale), with a decreasing craniocaudal gradient sparing the face. The attack recurred 2 months later with the same characteristics in addition to dysarthria but without apparent triggers.

Interictal electroencephalography (EEG) during wakefulness and sleep showed focal high‐amplitude sharp‐wave discharges, although the patient never had epileptic seizures. Brain magnetic resonance imaging showed no abnormalities.

A next generation sequencing panel was performed in a trio (proband and his parents) and showed a novel heterozygous de novo *AP2M1* (NM_004068.4):c.100G>A p.(Val34Ile) missense variant, whereas no relevant variants were found in the known causative genes of HM—*CACNA1A*, *ATP1A2*, and *SCN1A*. The variant is located in a strongly conserved amino acid residue in the functional N‐terminal domain of the AP2M1 protein (conservation scores phyloP100 = 9.374, supporting pathogenic > 7.52). The variant is absent from local and public databases (GnomAD, 1000 Genomes Project database, and ClinVar), and it is judged by prediction tools as “disease‐causing” by Mutation Taster, “benign” by PolyPhen 2, and “likely pathogenic” (6 points = 6Pathogenic‐0Benign [Pathogenic Moderate criterion 2 + Pathogenic Supporting criterion 2 + Pathogenic Strong criterion 2]) by VarSome, according to the American College of Medical Genetics and Genomics guidelines.

Despite the abundant literature on AP2 complex cellular functions, only two clinical studies discuss its pathogenic role in human diseases. A recurrent de novo variant (*AP2M1*:c.508C>T, p.Arg170Trp) was functionally demonstrated in four unrelated female individuals associated with GDD (4/4), autism spectrum disorder (2/4), and the electroclinical phenotype known as myoclonic–atonic seizures or Doose syndrome (3/4).^1^ Finally, a novel de novo heterozygous variant (*AP2M1*:c.73G>A, p.Gly25Arg) was found in a 3‐year‐old boy with GDD, macrocephaly, and multifocal epileptiform activity on EEG without epilepsy.^2^

We present a patient with ADHD, oppositional defiant disorder, and sporadic hemiplegic migraine associated with focal sharp‐wave discharges on EEG without epileptic seizures.

Analysis of the available clinical data confirms a prevalent pattern of cognitive and behavioral impairment in all patients. In addition, a subgroup of patients presents a specific epileptic pattern, whereas others present multifocal sharp‐wave discharges without epilepsy. Although a genotype/phenotype correlation might be a long shot, given the low number of patients reported so far, the phenotypes described appear to objectively segregate into two subtypes. The first, more severe, is characterized by “epileptic encephalopathy” related to the Arg170 variant located in the C‐terminal AP2M1 domain. The second, less severe, is characterized by “cognitive and behavioral deficit” associated with variants localized in the N‐terminal AP2M1 domain (Gly25, Val34).

The conformational change of AP2 complex, necessary for binding to endocytic signals, mostly involves the C‐terminal AP2M1 domain, whereas the N‐terminal AP2M1 domain does not show important conformational changes.^3^ This evidence could elucidate the variability of clinical features documented in the patients investigated.

We hypothesize that an altered quantity or function of the AP2M1 protein impairs the CME process, which is essential for the correct membrane transport of N‐methyl‐D‐aspartate, α‐amino‐3‐hydroxy‐5‐methyl‐4‐isoxazolepropionic acid, and γ‐aminobutyric acid receptors. Based on the experimental evidence, the dysregulation of the abovementioned receptors is considered to underlie behavioral and cognitive disorders. In addition, the dysregulation of N‐methyl‐D‐aspartate receptor is involved in cortical spreading depolarization/depression, which is the mechanism underlying HM. Therefore, we consider plausible, although not yet experimentally proven, a correlation between the proband phenotype and the AP2M1‐Val34Ile variant.

Recently, a small number of genes, different from those known to be implicated in familial HM, have been identified through next generation sequencing and whole‐exome sequencing analyses, but with a moderate functional effect size and therefore with uncertain evidence of HM causality.^4^ Furthermore, recent genome‐wide association studies have identified approximately 200 migraine susceptibility loci,^4^ one of which reported a shared genetic susceptibility between migraine and psychiatric disorders.^4^ Overall, migraine is a complex disorder in most cases caused by intricate genetics, ranging from the monogenic Mendelian model to gene–gene interaction with synergistic effect, and gene–environment interaction.

## AUTHOR CONTRIBUTIONS

**Giuseppe D. Mangano:** Conceptualization; writing – original draft; writing – review and editing; project administration; formal analysis. **Vincenzo Antona:** Data curation; formal analysis. **Giuseppe Santangelo:** Data curation; investigation; supervision. **Gabriele Di Pasquale:** Writing – review and editing; writing – original draft. **Jacopo Colella:** Writing – original draft; writing – review and editing. **Vincenzo Salpietro:** Conceptualization; supervision; writing – review and editing. **Giuseppa R. Mangano:** Data curation; formal analysis; writing – review and editing. **Vincenzo Raieli:** Data curation; formal analysis; investigation; writing – original draft; writing – review and editing.

## CONFLICT OF INTEREST STATEMENT

**Giuseppe D. Mangano**, **Vincenzo Antona**, **Giuseppe Santangelo**, **Gabriele Di Pasquale**, **Jacopo Colella**, **Vincenzo Salpietro**, **Giuseppa R. Mangano**, and **Vincenzo Raieli** declare no conflicts of interest.
